# Brown syndrome: clinical features and results of superior oblique tenotomy

**DOI:** 10.5935/0004-2749.20210021

**Published:** 2025-02-02

**Authors:** Ercan Ozsoy, Abuzer Gunduz, Ilknur Tuncer Firat, Murat Firat

**Affiliations:** 1 Department of Ophthalmology, Haseki Training and Research Hospital, University of Health Sciences, Istanbul, Turkey; 2 Department of Ophthalmology, Inonu University School of Medicine, Malatya, Turkey; 3 Department of Ophthalmology, Malatya Training and Research Hospital, Malatya, Turkey

**Keywords:** Adduction, Elevation, Hypotropia, Resolution, Tendon, Adução, Elevação, Hipotropia, Resolução, Tendão

## Abstract

**Purpose:**

This study was conducted to further de fine the specific clinical characteristics of patients with Brown syndrome and evaluate the outcomes of superior oblique tenotomy in its surgical management.

**Methods:**

A retrospective analysis of the medical charts of 45 patients with Brown syndrome was performed, which revealed that 11 patients underwent superior oblique tenotomy due to abnormal head posture and/or hypotropia and 1 patient underwent bilateral superior oblique tendon elongation with a silicone band due to abnormal head posture. In the last patient, silicone bands were removed at the postoperative 3rd month due to the lack of improvement in the abnormal head posture and the limitation of elevation in adduction. Simultaneous horizontal rectus muscle surgery was performed in four patients.

**Results:**

There was a predominance of female gender, right eye, congenital form, unilaterality, A-pattern, and an abnormal head posture type with a combination of chin up and head tilting. Bilateral form was observed only in female patients. Amblyopia was detected in two patients. Among patients aged >5 years, 40% had reduced stereopsis. Abnormal head posture was found in 60% of patients. More than half of them were diagnosed with a vertical and/or horizontal deviation. Tenotomy procedure eliminated the abnormal head posture in all patients and significantly improved the mean limitation of elevation in adduction and hypotropia (p=0.001, p=0.012). Two patients developed inferior oblique overaction in the operated eye. There was complete spontaneous resolution in two patients.

**Conclusions:**

The clinical features of patients with Brown syndrome in our study are considerably consistent with those of previous reports. The present study demonstrated the effectiveness of superior oblique tenotomy with less overcorrection in the surgical treatment of Brown syndrome.

## Abstract (pt)

**Objetivo:**

Definir mais detalhadamente as características clínicas específicas de pacientes com síndrome de Brown e avaliar os resultados da tenectomia do músculo oblíquo superior no manejo cirúrgico da síndrome de Brown.

**Métodos:**

Prontuários de 45 pacientes com síndrome de Brown foram analisados retrospectivamente. Onze pacientes submetidos à tenectomia do músculo oblíquo superior devido a postura anormal da cabeça ou a hipotropia e um paciente submetido ao alongamento bilateral do tendão do oblíquo superior com uma faixa de silicone devido a postura anormal da cabeça. Neste último paciente, a faixa de silicone foi removida no terceiro mês pós-operatório devido à ausência de melhora na postura anormal da cabeça e à limitação da elevação em adução. Quatro pacientes submeteram-se simultaneamente à cirurgia do músculo reto horizontal.

**Resultados:**

Houve predominância de sexo feminino, olho direito, forma congênita, acometimento unilateral, padrão em “A” e um tipo de postura anormal da cabeça combinando queixo elevado e inclinação da cabeça. A forma bilateral foi vista apenas em pacientes do sexo feminino. Foi constatada ambliopia em 2 pacientes. Dentre os pacientes acima de 5 anos de idade, 40% tinham estereopsia reduzida. Pos tura anormal da cabeça estava presente em 60% dos pacientes. Mais da metade dos pacientes foi diagnosticada com um desvio vertical, horizontal ou ambos. O procedimento de tenectomia eliminou a postura anormal da cabeça em todos os pacientes e melhorou significativamente a limitação média da elevação em adução e a hipotropia (p=0,001 e p=0,012). Dois pacientes desenvolveram hiperação do músculo oblíquo inferior no olho operado. Resolução completa ocorreu espontaneamente em 2 pacientes.

**Conclusões:**

O quadro clínico dos pacientes com síndrome de Brown no nosso estudo é bastante consistente com os relatos iniciais na literatura. Este estudo mostrou a eficácia da tenectomia do oblíquo superior, com menor hipercorreção no tratamento cirúrgico da síndrome de Brown.

## INTRODUCTION

Brown syndrome (BS) is characterized by limited active and passive elevation of the affected eye in adduction^(1,2)^. In addition to the limitation of elevation in adduction (LEA), patients with BS can manifest a widening of the palpebral fissure in adduction, divergence in upgaze, hypotropia in primary position, disfiguring downshoot during adduction, and abnormal head posture (AHP)^(3-5)^. BS can be induced by a number of causes, including anomalies of the superior oblique (SO) tendon or of the trochlea, inflammatory diseases such as rheumatoid arthritis and systemic lupus erythematosus, infection, trauma, neoplasm, and iatrogenic processes^(6-9)^.

Regarding the management of BS, mild and moderate cases can be observed for spontaneous resolution, but the healing process may take a long time. Surgical treatment is performed in severe cases. Patients with vertical deviation in the primary gaze position, AHP, significant diplopia, or disfiguring downshoot in adduction are candidates for surgical intervention^(10-12)^. Several surgical procedures have been introduced for BS treatment, including SO tenectomy, SO tenotomy, Z tenotomy, split tendon lengthening, and tendon lengthening using the silicone band, adjustable nonabsorbable sutures, or autogenous expanders^(6,13-16)^.

The purpose of this study was to describe the clinical features of patients with BS and evaluate our surgical results in patients with BS.

## METHODS

In this retrospective study, the medical records of patients who had been diagnosed with BS were reviewed. The study was performed at Inonu University Medical Faculty’s Ophthalmology Department between January 2012 and December 2017. An informed consent was obtained from all patients or their parents before conducting the study. The study protocol was approved by the Institutional Review Board and conducted according to the rules and regulations of the Declaration of Helsinki. Patients who had the clinical features of BS, including limitation of active elevation in adduction, AHP, hypotropia in primary position, or downshoot during adduction, were enrolled. Patients with any previous strabismus or orbital surgery, coexisting restriction other than limitation of elevation during adduction, incomplete records, and less than 6 months of follow-up were excluded from the study.

Based on visual acuity (VA) measurements performed using the Snellen or Lea chart, the amblyopic patients were determined. Amblyopia was defined as a best spectacle corrected visual acuity (BSCVA) of ≤20/30 and at least a difference of two lines between the amblyopic and normal eye of an individual. Stereopsis was measured using the Titmus stereoacuity test (Stereo Optical, Chicago, IL, USA) in compliant patients. AHP was measured using a goniometer on the vertical axis in the primary gaze position. Vertical and horizontal deviations in the primary position were measured using prism cover or Krimsky tests. LEA was recorded on a scale of 0 to -4, where 0, normal elevation; -1, elevation was 25% less than normal; -2, elevation was 50% less than normal; -3, elevation was 75% less than normal; and -4, the center of the pupil did not pass the imaginary line connecting the medial to lateral canthal angle^(4,12)^.

Indications for surgery included the presence of AHP and/or hypotropia in the primary position. In total, 11 patients underwent SO tenotomy and 1 patient underwent bilateral SO tendon elongation with a silicone band. A forced duction test was performed to confirm the presence of BS in all patients before the surgery. In 4 of 10 patients associated with horizontal deviations, concurrent horizontal rectus muscle surgery was performed.

### Surgical techniques

SO tenotomy: The SO tendon was isolated in the superotemporal quadrant and cut just medial to the superior rectus muscle. The forced duction test was repeated to determine whether there was complete improvement in limited elevation. The conjunctival incision was closed using 8-0 polyglactin sutures.

SO tendon elongation: After a conjunctival incision, the SO tendon was hooked on the nasal aspect of the superior rectus muscle. A small incision was performed through the tendon capsule to visualize the tendon fibers directly. Two 7-0 prolene sutures with spatulated needles were preplaced. The first suture was passed through the SO tendon 3 mm from the nasal border of the superior rectus muscle, and the second suture was placed in the same manner 2 mm nasal to the first suture. The SO tendon was cut between the preplaced sutures. The forced duction test was repeated to confirm whether the tendon has been completely transected. A 6 mm silicone band (No. 240) was then sutured between the cut ends of the tendon with the preplaced prolene sutures. The tendon capsule was closed over the silicone expander. The conjunctival wound was closed using 8-0 polyglactin sutures.

Statistical analysis was performed using the SPSS software version 22.0 (SPSS Inc., Chicago, IL, USA). Descriptive data were represented as mean ± standard deviation (mean ± SD), median (minimum-maximum), number (n), or percentage (%). The Shapiro-Wilk test was used to determine whether the data distribution is normal. Mann-Whitney U, Wilcoxon, and paired *t*-tests were used for data analysis. A p value of <0.05 was considered to be statistically significant.

## RESULTS

The median age of the patients included in this study was 62 months (range: 14-444 months). There were 28 females (62.22%) and 17 males (37.78%). The mean follow-up duration was 22.58 ± 16.25 months. A total of 43 patients (95.55%) had the congenital form of BS, and 2 (4.45%) had the acquired form. One of the acquired forms was caused by trauma, and the other one was caused by sinusitis. BS was unilateral in 40 patients (88.89%) and bilateral in 5 patients (11.11%). All patients with the bilateral form were female. In the unilateral patients, the right eye was affected in 22 patients (55%), and the left eye was affected in 18 patients (45%).

The median spherical equivalent was 0.50 diopter (D) and ranged from -18.50 to 4.50 D. In 2 patients (4.45%) younger than 36 months of age, the VA could not be mea sured. The mean BSCVA was 0.83 ± 0.27. Amblyopia was diagnosed in 2 patients (4.45%) and was associated with horizontal strabismus. One patient (2.22%) had vision loss due to degenerative myopia. Stereopsis measurements could be performed in 30 patients (66.67%) over 5 years of age, with 18 (60%) demonstrating stereopsis ranging from 40 to 100 seconds of arc and 12 (40%) demonstrating stereopsis ranging from 100 to 3000 seconds of arc. Stereopsis could be measured in 6 of 10 patients associated with horizontal deviations and ranged between 100 and 3000 seconds of arc.

Exotropia was diagnosed in 9 patients (20%), esotropia in 1 patient (2.22%), and hypotropia in 15 patients (33.33%) in the primary position. Two patients (4.45%) had inferior oblique overaction (IOOA) in the unaffected eye. The 2 patients with contralateral IOOA also had an AHP. Overall, AHP was observed in 27 patients (60%). Five patients (11.11%) showed an A-pattern, and 4 patients (8.89%) showed a V-pattern. The mean LEA of all patients was -2.74 ± 0.66.

Four patients (5 eyes; 1 patient had the bilateral form) with AHP, 4 with hypotropia, and 3 with AHP and hypotropia underwent SO tenotomy, and 1 patient with the bilateral form underwent bilateral SO tendon elongation with a silicone band due to AHP. In the last patient, the silicone expanders were removed at 3 months postoperatively because of a lack of improvement in AHP and LEA, and the ends of the SO tendon were released as done in the tenotomy procedure in 2 eyes of this patient. Four patients (8.89%) with exotropia underwent concurrent horizontal rectus muscle surgery.

Postoperatively, AHP was completely resolved in all patients, the mean LEA improved from -2.71 ± 0.73 to -0.43 ± 0.65 (p=0.001), and the mean hypotropia decreased from 12.17 ± 12.51 to 2.50 ± 4.73 prism diopters (PD) (p=0.012). Two of the 4 patients (50%) who underwent horizontal rectus muscle surgery had residual exotropia. In 2 patients (16.67%), IOOA was observed in the operated eye. Table 1 shows the clinical characteristics of the patients who underwent surgery and the surgical procedures used.

**Table 1:** Data of patients who underwent surgery

Case no Gender		Eye	Age at surgery (month)	Preop. LEA	Preop. devi. (PD)	Preop. AHP (+/-)	Surgical procedure	Postop. LEA	Postop. devi. (PD)	Postop. AHP (+/-)	Sec. IOOA
1	F	R+L	251	-3	0	+	Bil.ten. elon.+ Bil.SOT	-1	5 hypo.	-	-
2	F	R	124	-2	0	+	RSOT	0	0	-	+3
3	M	R	57	-2	10 hypo.	+	RSOT	0	0	-	-
4	F	L	72	-1	0	+	LSOT	0	0	-	+1
5	F	L	110	-3	25 hypo.	-	LSOT	0	4 hypo.	-	-
6	F	L	47	-3	16 XT	+	LSOT + LLR recess. 6 mm	0	6 XT	-	-
7	F	L	90	-4	30 hypo.+ 30 XT	-	LSOT + LLR recess. 8 mm + LMR resec. 4 mm	0	0	-	-
8	M	L	359	-2	25 hypo.+ 16 XT	-	LSOT + LLR recess. 4 mm + LMR resec. 4 mm	-1	5 hypo.	-	-
9	F	L	100	-3	8 hypo.	+	LSOT	0	0	-	-
10	F	R+L	203	-3	0	+	Bil.SOT	0	0	-	-
11	F	R	331	-3	20 hypo.+ 35 XT	-	RSOT + RLR recess. 6.5 mm + RMR resec. 5 mm	-1	10 XT	-	-
12	M	R	60	-3	28 hypo.	+	RSOT	-2	16 hypo.	-	-

Preop.= preoperative; Postop.= postoperative; AHP= abnormal head posture; LEA= limitation of elevation in adduction; Sec.= secondary; IOOA= inferior oblique overaction; PD= prism diopter; hypo.= hypotropia; XT= exotropia; LSOT= left superior oblique tenotomy; RSOT= right superior oblique tenotomy; Bil. ten. elon.= bilateral tendon elongation; mm= milimeters; recess.= recession; resec.= resection; RLR= right lateral rectus; LLR= left lateral rectus; RMR= right medial rectus; LMR= left medial rectus.

In 15 (45.45%) of 33 patients who did not undergo surgery, there was partially spontaneous recovery in patients with AHP, hypotropia, or LEA. Complete spontaneous resolution was observed in 2 patients (6.06%).

## DISCUSSION

This retrospective study evaluated 45 patients with BS and detected a preponderance for the female gender, right eye involvement, congenital form, and unilaterality. In 126 cases collected by Brown, the disorder was found to more commonly affect the female gender (58.73%) and the right eye (61.90%)^(17)^. Eustis et al. reported 70% involvement of the right eye and no gender dominance in 30 patients^(18)^. In another study conducted by Cho et al., 60% of the patients were females, and 60% of the affected eye was the left eye^(8)^. These previous studies indicate that gender and eye predilections in this disorder have not yet been determined precisely. Bilateral involvement in BS has been reported to be approximately 10%^(19)^. In the present study, bilateral involvement was detected in 11.11% of the cases, which is consistent with the reported rate^(19)^.

We found no previous study that examined the refractive status of patients with BS, except for the original series of Brown’s 126 cases^(17)^. Brown reported no significant importance of refractive errors^(17)^. In our study, the spherical equivalent of patients ranged from -18.50 to 4.50 D, with a median of 0.50 D. Amblyopia was found in 2 of 45 patients (4.45%) and was associated with horizontal strabismus in these 2 patients. Wright diagnosed amblyopia in 3 of 85 patients (3.52%), with 2 cases of anisometropic amblyopia and 1 case of strabismic amblyopia^(20)^. Brown reported no significant frequency of amblyopia in his series of 126 cases^(17)^. On the other hand, Clarke and Noel reported amblyopia in 7 of 28 patients (25%), a relatively high frequency of ambliyopia in patients with BS^(21)^. We believe that the differences in the frequency of amblyopia are related to the small number of patients in each study, as well as the differences in measuring VA in each study.

Wright reported a stereopsis range between 40 and 3000 seconds of arc in 21 of 26 patients (81%) with congenital BS and in 26 of 36 patients (72%) with acquired BS^(20)^. In the study reported by Dawson et al., stereopsis was measured in 29 of 32 patients (90.62%) with congenital BS and was reported to range from gross stereopsis (1680 arcsec) to 40 seconds of arc^(3)^. In our study, stereopsis could be measured in 30 patients (66.67%) older than 5 years of age, and 18 (60%) patients demonstrated stereopsis between 40 and 100 seconds of arc, and 12 (40%) demonstrated stereopsis between 100 and 3000 seconds of arc.

Wright reported that 15 of 38 patients (39%) with congenital BS had orthotropia, and 6 (18%) had horizontal strabismus^(20)^. Dotan et al. reported horizontal strabismus in 9 of 16 patients (56.25%) who underwent surgery due to congenital BS, and 8 of them (50%) had an exodeviation, and 1 (6.25%) had esotropia^(2)^. Stager et al. detected horizontal deviations in 5 of 19 patients (26.31%) who underwent surgery due to congenital BS. They diagnosed esodeviations in 4 patients (21.05%) and exodeviation in 1 patient (5.26%)^(22)^. In another study conducted by Cho et al., there was a higher percentage of horizontal misalignment in patients who underwent surgery due to BS. They found horizontal deviations in 13 of 15 patients (86.67%) with BS, with exodeviations in 7 patients (46.67%) and esodeviations in 6 patients (40%)^(8)^. In the present study, 20 patients (44.45%) had orthotropia and 25 (55.55%) had a vertical deviation and/or horizontal deviation. We detected horizontal strabismus in 10 patients (22.22%), exotropia in 9 patients (20%), and esotropia in 1 patient (2.22%). Our results regarding the frequency of orthotropia and horizontal deviation are comparable with those reported by Wright ^(20)^, which was methodologically similar to our study. The other three studies^(2,8,22)^ included only patients who underwent surgery.

Contralateral IOOA has been reported to be associated with unilateral BS. Limitation of elevation in adduction due to BS results in increased innervation to the ipsilateral superior rectus muscle and the contralateral inferior oblique muscle based on the Hering’s law, which postulates that both eyes receive equal innervation. The affected eye is limited, but the contralateral eye overelevates in adduction. The contralateral IOOA caused by limited elevation of the BS eye in adduction is considered as a pseudo-overaction^(20,23)^. Wright reported contralateral IOOA in 5 of 85 patients (5.88%) with BS^(20)^. In our study, we observed contralateral IOOA in 2 patients (4.45%). Thus, our finding on the frequency of contralateral IOOA is similar to that reported by Wright^(20)^.

In the 126 cases reported by Brown, AHP was detected in 38 cases (34.54%)^(17)^. Wright did not focus on AHP in his study that included 85 cases^(20)^. Dawson et al. reported AHP in 19 of 32 patients (59.37%) with congenital BS^(3)^. We detected AHP in 27 patients (60%). The most common AHP type was a combination of chin up and head tilting with a ratio of 20%. The rate of AHP found in our study is consistent with the outcome of the study of Dawson et al.^(3)^, which consisted of a similar sample.

The literature consists of two studies with a large number of cases reporting the clinical features of BS. One of them, Brown’s 126 cases, did not focus on the pattern of strabismus^(17)^. The other one, Wright’s 85 cases, reported the V-pattern in 24 cases (28.23%) and the A-pattern in 1 case (1.17%)^(20)^. We diagnosed the A-pattern in 5 patients (11.11%) and the V-pattern in 4 patients (8.89%). In our study, 3 of 5 patients with the A-pattern had reduced stereopsis (between 100 and 3000 seconds of arc), and the remaining 2 patients were too young to evaluate stereopsis. Of those patients with the A-pattern, 4 had horizontal strabismus and the other one had no horizontal misalignment. In addition, 2 of 12 patients who underwent surgery had an A-pattern, and all patients who underwent surgery had a positive intraoperative forced duction testing, which confirmed the presence of BS. Therefore, we believe that at least the 2 patients with the A-pattern in our surgical group had no SO overaction or inferior oblique palsy. Till date, there is no clear explanation regarding the etiology of A-pattern strabismus. Over the years, several theories have been suggested to clarify the etiology of the A-pattern, including oblique muscle dysfunction^(24)^, rectus muscle pulley abnormalities^(25)^, horizontal rectus muscle dysfunction^(26)^, and paresis of the vertical rectus muscle^(27)^. However, the oblique muscle dysfunction is considered to be the most common cause of A-pattern strabismus^(28)^. The A-patterns found in our study may be caused by other possible etiological mechanisms other than the oblique muscle dysfunction. Further studies are required to explain the high frequency of the A-pattern in our study.

In the surgical treatment of BS, the primary focus has been on the weakening/lengthening of the SO tendon^(29)^. SO tenotomy, one of the commonly used procedures, is technically simple and requires a short operation time, but it carries a risk for the possibility of iatrogenic SO muscle palsy with overcorrection^(12,14)^. The SO tenectomy procedure, in which a portion of the tendon is removed, has been found to be associated with a high incidence of iatrogenic SO paralysis^(30)^. To overcome the occurence of SO palsy after tenetomy or tenectomy, SO tendon spacer techniques have been introduced. All spacer techniques are more difficult and time-consuming than tenotomy. The use of silicone band expanders has been reported to be associated with several complications, such as SO paresis, sterile orbital cellulitis, inflammation, foreign body sensation, extrusion of the silicone band, and adhesions^(20,31-33)^. Recently, adjustable nonabsorbable suture spacers have increasingly been used for the weakening SO tendon with minimum side effects^(12,16,34)^. In recent years, autologous spacers such as facia lata and palmaris longus tendon have also been used for lengthening the SO tendon with minimal complications^(13,35)^.

**Figure 1:**
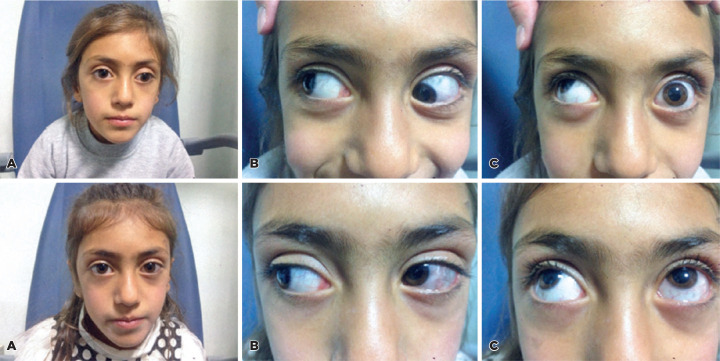
A patient (seventh case in table 1) with Brown syndrome in the left eye,preoperative (above); left hypotropia and exotropia in the primary position (A), limited elevation of the left eye in adduction (B), no movement of the left eye in upgaze (C); postoperative (below); orthotropia in the primary position (A), relieved elevation of the left eye in adduction (B) normal movement of the left eye in upgaze (C).

In the present study, we performed bilateral SO tendon elongation using a silicone band in 1 patient (the first case of our series) with bilateral BS due to AHP. As there was no improvment in AHP and LEA at the postoperative 3rd month, the silicone bands were removed in both eyes and the cut ends of the tendon were released as done in the tenotomy procedure. SO tenotomy was performed in the remaining 11 consecutive patients because of our disappointment with the first case. The tenotomy procedure eliminated AHP in all patients and significantly reduced the mean LEA and hypotropia (Figure 1). Five patients (41.66%) had preoperative and postoperative stereopsis testing records, of whom 2 (16.66%) had an improved stereopsis and 3 (25%) had a stable stereopsis. Crawford performed SO tenotomy in 16 patients with true BS and found excellent results in 9 patients (56.25%), good results in 3 patients (18.75%), improved results in 3 patients (18.75%), and unimproved result in 1 patient (6.25%). The cause of failure in the last patient was reported to be due to a missed tendon during the operation. Crawford reported the results of SO tenotomy as the best^(6)^. In the study conducted by Wright, 6 patients with congenital BS underwent SO tenotomy, and with a mean 4 months of postoperative follow-up, 5 of 6 patients (83.33%) were reported to have a vertical deviation greater than 5 PD, 2 of 6 patients (33.33%) had a significant residual restriction, and 3 of 6 patients (50%) had an overcorrection^(20)^. Wright reported the results of SO tenotomy as poor^(20)^. Cho et al. performed SO tenotomy in 4 patients with BS and reported that the mean vertical deviation decreased from 18.00 ± 16.08 to 3.00 ± 3.82 PD, and LEA completely resolved in 1 patient (25%) and improved in 2 patients (50%)^(8)^.

In the present study, we found that 2 patients (16.66%) had IOOA in the operated eye in the postoperative follow-up ranging from 6 to 51 months (median, 13 months). Of these patients with postoperative ipsilateral IOOA, 1 had a +3 IOOA in the postoperative 5th month and underwent inferior oblique anterior transposition surgery. The other patients who had +1 IOOA was followed up. Crawford reported secondary IOOA in 7 of 16 patients (43.75%) who underwent SO tenotomy due to true BS and 3 of these 7 patients required further surgery^(6)^. Wright reported secondary IOOA in 3 of 6 patients (50%) who underwent SO tenotomy due to congenital BS and believed that these 3 overactions of the inferior oblique were due to postoperative SO paresis^(20)^. In the study performed by Cho et al., secondary IOOA was found in 1 of 4 patients (25%) who underwent SO tenotomy due to BS^(8)^.

Spontaneous resolution has been reported to occur in patients with BS, but its incidence remains unknown. Among Brown’s 126 cases with SO tendon sheath syndrome, spontaneous recovery was reported in 9 cases (7.14%), which were classified as simulated sheath syndrome^(17)^. Dawson et al. monitored 32 patients with congenital BS for a mean follow-up of 4 years and reported a spontaneous recovery of ocular motility in 24 patients (75%) and an improvement of stereopsis in 11 patients (34.37%)^(3)^. In the study reported by Wright, 5 of 85 patients (5.88%) with BS experienced spontaneous recovery to within -1 LEA in a period of 6 to 48 months. Moreover, all spontaneous resolutions were observed in patients with acquired BS^(20)^. In our study, 15 of 33 patients (45.45%) had a spontaneous improvement in one of the AHP, hypotropia, or LEA without any intervention over a period of 9 to 75 months. Complete spontaneous resolution was observed in 2 patients (6.06%) in our study.

The present study has some limitations because of its retrospective design. First, the number of cases included in the study was low. Moreover, overcorrection, a well-known complication of SO tenotomy, developed in 2 cases after the tenotomy procedure. If we had used the adjustable nonabsorbable suture procedure that was becoming popular at that time, we might not have encountered this complication. We also had no chance of including a control group to compare our surgical results. Finally, the minimum postoperative follow-up period was short (6 months). It is known that the effect of SO weakening procedures on the elevation in adduction tends to increase over time, probably resulting in SO paresis. Therefore, a longer postoperative follow-up is required to evaluate the long-term effects of SO tenotomy.

In conclusion, most of the clinical features of patients with BS in this study were nonconflicting with the findings from early reports, except for refractive errors and A- pattern strabismus. The present study also demonstrated that SO tenotomy is an effective option in the surgical treatment of BS with less overcorrection and additional surgery. However, our surgical results must be confirmed by further studies with more cases and longer follow-up.
